# Lyme Borreliosis with Scalp Eschar Mimicking Rickettsial Infection, Austria

**DOI:** 10.3201/eid2609.191256

**Published:** 2020-09

**Authors:** Mateusz Markowicz, Anna-Margarita Schötta, Michiel Wijnveld, Gerold Stanek

**Affiliations:** Medical University of Vienna, Vienna, Austria

**Keywords:** Lyme borreliosis, eschar, Borrelia burgdorferi, rickettsiosis, scalp eschar and neck lymphadenopathy, SENLAT, bacteria, Lyme disease

## Abstract

We report on a patient in Austria with scalp eschar and neck lymphadenopathy. Rickettsial etiology was excluded by culture, PCR, and serologic tests. *Borrelia afzelii* was identified from the eschar swab by PCR. Lyme borreliosis can mimic rickettsiosis; appropriate tests should be included in the diagnostic workup of patients with eschars.

Scalp eschar and neck lymphadenopathy (SENLAT) is frequently caused by *Rickettsia slovaca* and *R. raoultii*. Reports on other etiologic agents have expanded the knowledge about the cause of this clinical entity (*1**,**2*). In some cases the causative agents remain undetermined. We report a case of SENLAT in a patient from Austria with serologic and molecular evidence for infection with *Borrelia burgdorferi* sensu lato (s.l.).

## The Patient

A 61-year-old woman was referred to the outpatient department of the Institute for Hygiene and Applied Immunology, Medical University of Vienna (Vienna, Austria), because of fatigue, muscle pain, and elevated body temperature (37.7°C) of 1 week’s duration. Before the onset of symptoms she reported having a tick bite on her head, and she noticed a palpable crust at the site of the bite. The patient was healthy, aside from having arterial hypertension and hypothyroidism, and she reported having contact with ticks and horses. She stated that ticks were highly abundant in her garden and frequently infested the horses. The physical examination revealed a crusty lesion measuring 0.5 cm, compatible with an eschar (Figure); lymph nodes in the neck were enlarged. The clinical picture and the history of tick exposure suggested a rickettsial infection.

**Figure:**
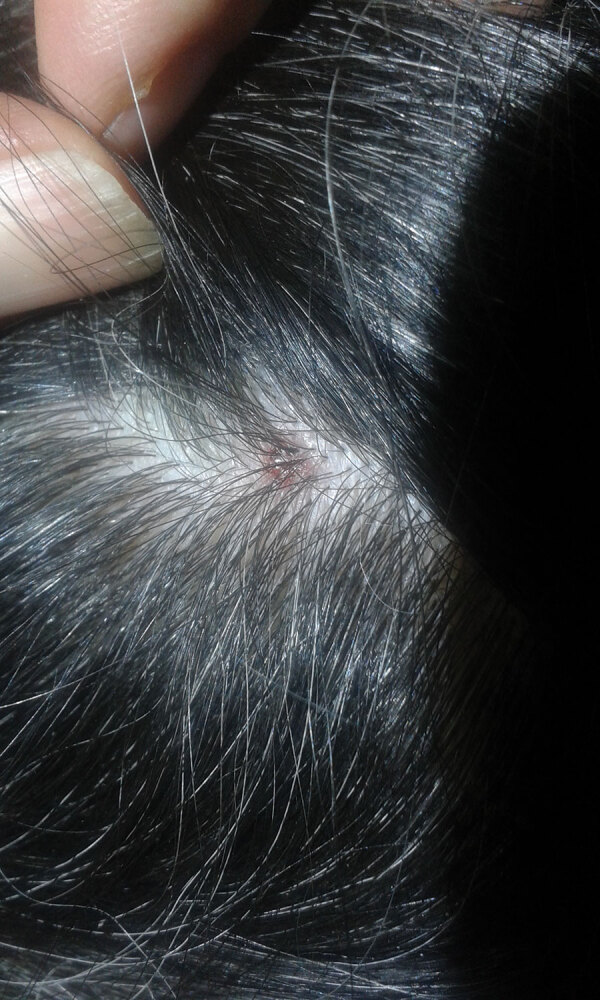
Scalp eschar of a woman in Austria who was found to be infected with *Borrelia afzelii*.

Basic laboratory tests showed cell counts and liver function parameters within reference ranges, elevated lactate dehydrogenase (341 U/L, reference <247 U/L), and moderately elevated C-reactive protein (0.59 mg/dL, reference <0.5 mg/dL). Serologic tests for tickborne diseases using Anti-*Borrelia* plus VlsE ELISA for IgG and Anti-*Borrelia* ELISA for IgM (Euroimmun, https://www.euroimmun.com), *Rickettsia* IFA IgG (Focus Diagnostics, https://www.focusdx.com), and Weil-Felix agglutination assay (DiaMondial, http://www.diamondial.com) all showed results below the cutoff levels and were interpreted as negative. In addition, we obtained material from the scalp eschar for PCR and culture by removing the crust with sterile tweezers. We used parts of the crust for testing, together with a swab taken directly from the skin underneath the crust. We isolated DNA from the swab and crust parts using the PeqGOLD Tissue DNA Mini Kit (Peqlab, https://de.vwr.com/cms/life_science), according to the manufacturer’s instructions. We used real-time PCR to target the *gltA* gene of *Rickettsia* spp. (*3*). We also attempted to culture *Rickettsia* spp. as previously described (*4*). We placed tissue from the eschar site directly into a tick cell-culture vial containing a layer of BME/CTVM2 cells. The cell line was provided by Lesley Bell-Sakyi, The Tick Cell Biobank, Institute of Infection and Global Health, University of Liverpool (Liverpool, UK), and cultivated as described previously (*5**,**6*). We supplemented the L-15 medium (Leibovitz’s L-15 medium; GIBCO, Thermo Fisher Scientific, https://www.thermofisher.com) with an antibiotic–antimycotic mixture (GIBCO) to prevent unwanted contamination of bacterial or fungal origin. We prepared cytocentrifuge smears weekly from culture samples (»100 µL) and stained them using the Shandon Kwik-Diff kit (Thermo Fisher Scientific).

The rickettsial PCR was negative, and cultivation was not successful. To confirm the negative culture, we took samples containing supernatant and cells after 2 months’ incubation and tested them with the aforementioned real-time PCRs for *B. burgdorferi* s.l. and *Rickettsia* spp. but also received negative results. At the patient’s initial admission, we started treatment with doxycycline 200 mg for 10 days. and the patient reported a rapid improvement of her symptoms.

Three weeks after we began treatment, we repeated the serologic tests for the respective tickborne pathogens and measured high concentrations of antibodies against *B. burgdorferi* s.l.: IgG 44 U/mL (positive cutoff >22 U/mL) and IgM >200 U/mL (positive cutoff >22 U/mL). We used an immunoblot (Anti-*Borrelia* Euroline-RN-AT, Euroimmun) as a confirmatory test: for IgM, we observed strong reactivity for antigens p41, p39, and OspC; for IgG, we found strong reactivity for p41 and OspC and weak reactivity for VlsE. Levels of antibodies against *Rickettsia* spp. remained negative.

These findings confirmed that the patient had an infection with *B. burgdorferi* s.l.; therefore, we further analyzed the DNA extract from the swab to search for borrelial DNA. We used 2 real-time PCRs based on the 16S rRNA gene (*7*) and the flagellin gene (*3*); the 16S PCR gave a positive result. We used a nested PCR targeting the 5S–23S intergenic spacer region (*8**,**9*) for genospecies identification and purified the obtained amplicon using the QIAGEN Gel Extraction Kit (https://www.qiagen.com) before bidirectional sequencing (Microsynth, https://www.microsynth.ch). Comparison with known sequences available at the National Center for Biotechnology Information (https://blast.ncbi.nlm.nih.gov/Blast.cgi) yielded a 100% match to various *B. afzelii* strains.

## Conclusions

The most common skin manifestation of Lyme borreliosis is solitary erythema migrans (EM) (*10*); multiple EM, borrelial lymphocytoma, and acrodermatitis chronica atrophicans occur less frequently. Eschar formations are local necrotic skin alterations covered by a crust and occur at the site of a tick bite after infection with tickborne rickettsial species. Enlargement of local lymph nodes, skin rash, and malaise are additional symptoms that may be observed (*11*). Diagnosis for patients with EM is based on the clinical picture only; no laboratory support is needed in most cases. *Rickettsia* spp. can be easily identified by PCR, either from skin tissue obtained from the eschar or by taking a swab from the same site for PCR testing. Serologic tests may be helpful if PCR is not available. A 2-step increase of the antibody titer in consecutive samples in an immunofluorescence assay is needed for confirmation of infection. Treatment with doxycycline is effective for both diseases.

*Ixodes ricinus* ticks are capable of transmitting several pathogenic species of the *B. burgdorferi* s.l. complex; it is the most common tick species that feeds on humans in central Europe. *B. afzelii* has been recognized as the most frequent genotype associated with EM in Europe. In a study in Slovenia, 89% of *Borrelia* species detected from skin biopsies of EM patients were *B. afzelii* (*12*). Similarly, *B. afzelii* was the most prevalent *Borrelia* species found in ticks in Austria (*13*). In a recent study we showed that 56% of all *I. ricinus* ticks positive for *B. burgdorferi* sl harbored *B. afzelii* (*13*). The range of tick vectors of *Rickettsia* spp. is much more diverse; *Dermacentor reticulatus* and *D. marginatus* ticks are known to transmit *R. slovaca* and *R. raoultii* (*11*). We asked the patient to collect ticks from the area where she lives for identification of the tick species abundant there. The morphological examination of ticks collected from vegetation revealed only the presence of *I. ricinus* ticks; thus, it is unlikely that a species of *Dermacentor* tick transmitted the infection.

Identification of *B. afzelii* from the eschar of our patient was unexpected. Ni et al. have shown that eschar formations can occur after borrelial infection in patients with concomitant EM (*14*); however, it is not known whether those patients were also infected with *Rickettsia* spp. A molecular identification of *B. burgdorferi* s.l. in a tick from a patient with SENLAT has been reported (*2*), and our case study provides clear evidence that the spirochete can be detected directly in the scalp eschar. Moreover, we are not aware of any other report of molecular identification of *B. afzelii* from a skin swab and superficial skin material. Inclusion of *B. burgdorferi* in the differential diagnosis of patients with SENLAT appears justified, particularly if testing for *Rickettsia* spp. is not successful.
